# Single-cell trajectories of melanoma cell resistance to targeted treatment

**DOI:** 10.20892/j.issn.2095-3941.2021.0267

**Published:** 2021-10-01

**Authors:** Maria Schmidt, Lena Sünke Mortensen, Henry Loeffler-Wirth, Corinna Kosnopfel, Knut Krohn, Hans Binder, Manfred Kunz

**Affiliations:** 1 Interdisciplinary Centre for Bioinformatics, University of Leipzig, Leipzig 04107, Germany; 2 Department of Dermatology, Venereology and Allergology, University of Würzburg, Würzburg 97074, Germany; 3 Core Unit DNA Technologies, Medical Faculty, University of Leipzig, Leipzig 04103, Germany; 4 Department of Dermatology, Venereology and Allergology, University of Leipzig Medical Center, Leipzig 04103, Germany

**Keywords:** Melanoma, single-cell transcriptome sequencing, treatment response

## Abstract

**Objective:**

Cellular heterogeneity is regarded as a major factor affecting treatment response and resistance in malignant melanoma. Recent developments in single-cell sequencing technology have provided deeper insights into these mechanisms.

**Methods:**

Here, we analyzed a *BRAF*^V600E^-mutant melanoma cell line by single-cell RNA-seq under various conditions: cells sensitive to BRAF inhibition with BRAF inhibitor vemurafenib and cells resistant to BRAF inhibition with vemurafenib alone or vemurafenib in combination with the MEK1/2 inhibitors cobimetinib or trametinib. Dimensionality reduction by t-distributed stochastic neighbor embedding and self-organizing maps identified distinct trajectories of resistance development clearly separating the 4 treatment conditions in cell and gene state space.

**Results:**

Trajectories associated with resistance to single-agent treatment involved cell cycle, extracellular matrix, and de-differentiation programs. In contrast, shifts detected in double-resistant cells primarily affected translation and mitogen-activated protein kinase pathway reactivation, with a small subpopulation showing markers of pluripotency. These findings were validated in pseudotime analyses and RNA velocity measurements.

**Conclusions:**

The single-cell transcriptomic analyses reported here employed a spectrum of bioinformatics methods to identify mechanisms of melanoma resistance to single- and double-agent treatments. This study deepens our understanding of treatment-induced cellular reprogramming and plasticity in melanoma cells and identifies targets of potential relevance to the management of treatment resistance.

## Introduction

Malignant melanoma is a highly aggressive tumor with a well-established genetic background^1^. The most prevalent mutation in melanoma is *BRAF*^V600E^ (Val600→Glu600), which activates the mitogen-activated protein kinase (MAPK) pathway and can be targeted by small molecule inhibitors^2^. Currently, targeted treatment of metastatic melanoma uses 1 of the 3 BRAF inhibitors vemurafenib, dabrafenib, or encorafenib in combination with inhibitors of downstream targets such as mitogen-activated extracellular signaling-regulated kinases (MEK1/2)^2^. These combinations have significantly improved 5-year overall survival rates, which now exceed 50%^3^. Despite this, the vast majority of patients ultimately develop a secondary resistance^4^.

Tumor heterogeneity is a well-known phenomenon and is regarded as a major driver of treatment resistance in many cancers, including malignant melanoma^5,6^. The advent of single-cell sequencing technology has provided unprecedented resolution of this heterogeneity, and permitted detailed studies in a range of different cancers^7^. In the first landmark study on melanoma single-cell transcriptomes, Tirosh et al.^8^ analyzed 19 samples from primary melanomas and melanoma metastases. Transcriptional heterogeneity was defined in terms of gene expression patterns in both time and space, including different cell cycle and developmental states as well as the spatial contexts of the local microenvironment. Importantly, a drug-resistance program (MITF-low/AXL-high signature) was found in a small number of cells in samples that were generally regarded to be drug-sensitive (MITF-high/AXL-low), suggesting that latent treatment resistance programs were present in these samples. Mapping the gene expression patterns of non-melanoma cells onto melanoma sequencing data of the Cancer Genome Atlas Network has identified different types of tumor clusters on the basis of their inferred cell type composition^8^.

In an earlier study from our own group, self-organizing maps (SOMs) identified sub-populations of melanoma cells, based on gene expression patterns of cellular proliferation, oxidative phosphorylation, pigmentation, and stroma^9^. The results revealed that patterns affecting cellular proliferation were associated with the shortest overall survival in a clinical study when comparing these data with published gene expression profiles from patient biopsies^10^. Furthermore, proliferation and pigmentation gene signatures in primary melanomas were characteristic of a class of melanomas with poorer overall survival^11^.

In the present study, single-cell transcriptomic patterns were analyzed in A375 melanoma cell cultures under targeted treatment with MAPK inhibitors to identify transcriptomic patterns of developing treatment resistance. We applied a variety of bioinformatics methods including t-distributed stochastic neighbor embedding (tSNE) and SOM to visualize expression landscapes before and after treatment as well as pseudotime and RNA velocity analysis to infer paths of developing treatment resistance on a single cell level. We demonstrated that developmental trajectories showed transitions from a neural crest-like state to a more proliferative one, followed by states exhibiting signatures of MAPK pathway reactivation and pluripotency, suggesting a high degree of plasticity of cellular programs after treatment.

## Materials and methods

### Melanoma cell culture

The *BRAF*^V600E^-mutant melanoma cell line A375 was maintained under standard conditions in RPMI medium with 10% fetal calf serum and 1% penicillin/streptomycin. The identity of the A375 cells was confirmed before starting the experiments. Cells were made resistant to vemurafenib or a combination of vemurafenib with cobimetinib or trametinib (MEK1/2 inhibitor) by treating cells for 10–14 days with half of the previously determined IC50 concentrations. This resulted in an increase of IC50 mean values for vemurafenib from 3.06 µM for sensitive (S) cells to 13.94 µM for single-agent-resistant (vemurafenib; RV) cells and to 39.24 µM for double-agent (vemurafenib plus cobimetinib; RVC) and 15.96 (vermurafenib plus trametinib; RVT) cells. Values for cobimetinib increased from 0.55 µM for S cells to 9.18 µM for RVC cells and for trametinib from 0.78 µM for S cells to 9.82 µM for RVT cells. Inhibitors were removed 24 h before starting single-cell analyses to avoid direct toxic effects of substances on cell cultures. Chemical inhibitors were purchased from Biozol (Munich, Germany; vemurafenib, SEL-S1267; cobimetinib, SEL-S8041; trametinib, SEL-S2673).

### Single-cell RNA-seq (scRNA-seq)

Cell suspensions were generated by washing melanoma cells with phosphate-buffered saline then trypsinized with TripLE Xpress (Invitrogen, Darmstadt, Germany) for 5 min. The reaction was stopped by adding media containing fetal calf serum (FCS). Cell viability was determined by trypan blue staining (Sigma Aldrich, Munich, Germany) and always exceeded 95%. Single melanoma cells from short-term cultures were captured on a 10× Genomics Chromium Controller^®^ (10× Genomics, Pleasanton, CA, USA) according to the manufacturer’s instructions, with 10,000 cells being loaded into a channel of the Chromium system using the v2 single-cell reagent kit (10× Genomics^®^). Following capture and lysis, cDNA was synthesized and amplified for 12 cycles. The amplified cDNA was used to construct Illumina sequencing libraries according to the conditions described in the Chromium User Guide^®^ (10× Genomics). Briefly, blunt-end repair was followed by adapter ligation. Sample indexes were added during PCR amplification. The barcoded libraries were purified with SPRI beads and quantified using the Library Quantification Kit – Illumina/Universal (KAPA Biosystems; Merck KGaA Darmstadt, Germany) according to the manufacturer’s instructions. Correct size distribution of the library DNA was confirmed using the Fragment Analyzer (Agilent, Santa Clara, CA, USA). Sequencing of 2 × 150 bp was performed with an Illumina NextSeq 550 sequencer (Illumina, San Diego, CA, USA). Demultiplexing was done using bcl2fastq v2.20.0.422 (Illumina).

### Pre-processing and SOM analysis of scRNA-seq data

**Figure 1:**
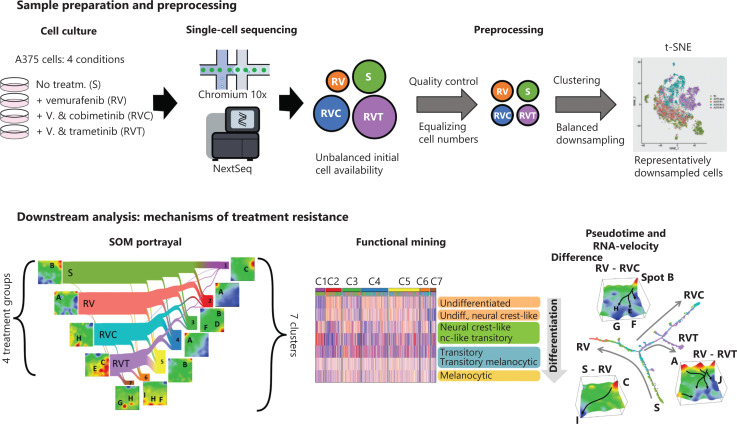
Overview of the experimental and analytical workflow. A375 melanoma cells were cultured in the presence of various mitogen-activated protein kinase pathway inhibitor drugs and analyzed by single-cell RNA-seq. After quality control, cell numbers were balanced and downscaled for further analysis. t-Distributed stochastic neighbor embedding analysis assigned cells to 7 transcriptional states distributed over the treatment groups. The underlying transcriptional landscapes were visualized using self-organizing map portraits. The downstream analysis included functional data mining by gene set analysis, comparison with previous melanoma studies, pseudotime, and RNA velocity analysis.

scRNA-seq data were further pre-processed using 10× Genomics software Cell Ranger^®^ (https://support.10xgenomics.com) and the R package Seurat (https://satijalab.org/seurat/). For subsequent transcriptional analysis, we applied oposSOM software^12–15^. Pre-processing of expression values included log-transformation and centralization of expression data. Cell numbers varied between treatment groups: 5,282 sensitive/untreated cells (S), 2,004 cells resistant to single-drug treatment with vemurafenib (RV), 9,266 cells resistant to combined treatment with vemurafenib and cobimetinib (RVC), and 10,427 cells resistant to combined treatment with vemurafenib and trametinib (RVT; see **Figure 1** for an overview). After quality control to remove dead and doublet cells, technical bias was removed by batch correcting the data using the IntegrateData protocol in the Seurat package. Then, cell numbers were equalized by random selection of 2,000 cells per condition to ensure equal weighting of treatment conditions in downstream analyses. To optimize the runtime of the oposSOM software, we sought to downscale the cell numbers while preserving the intrinsic heterogeneity of the samples. For this purpose, we employed Louvian clustering to group the cells in 27 clusters, and randomly selected 20% of cells per cluster and treatment group. Overall, 1,800 representative cells (out of originally 26,979) remained for SOM analysis. Note that the number of clusters used for segmentation (*n* = 27) is not limiting, insofar as it clearly exceeds the number of treatment groups (*n* = 4) and intrinsic clusters (*n* = 7).

We employed the oposSOM software to analyze transcriptome data *via* SOMs, a neural network machine learning technique previously described^14,15^. Briefly, it transforms the high-dimensional expression data of *n* = 28,065 gene transcripts into K = 3,600 metagenes. Metagenes were arranged in a 60×60 grid, and due to the self-organizing properties of the SOM, metagenes with similar expression profiles cluster together. Similarly, genes with similar expression profiles group together in the same or closely located regions of the grid. SOM analysis thus visualizes the individual transcriptome landscape of each cell, its “SOM portrait”, by coloring overexpressed and underexpressed metagenes in red and blue, respectively.

Clustering the expression portraits of all cells resulted in 7 clusters (C1–C7), each referring to cells with similar expression patterns. Mean SOM portraits of each cluster were obtained by averaging the portraits of all cells within clusters. The so-called spots of overexpressed metagenes were identified by applying a top 90% quantile criterion for metagene expression. Spots contained 162–653 genes. The functional context of each spot and cell cluster was determined using DAVID (https://david.ncifcrf.gov) and gene set analysis as implemented in oposSOM^15^.

### Similarity, pseudotime, and RNA velocity analysis

Similarities between the transcriptomes of melanoma cells were visualized using tSNE applied to the cells in all treatment conditions. Pseudotime analysis was performed using *monocle*^16^. and *URD* as independent methods^16,17^. Pseudotime analysis arranges cells according to similarities of their transcriptomes along a branched-tree structure inferring potential trajectories of cell development, e.g., under the effect of treatment. RNA velocities were calculated as rate of gene expression change using the ratio of unspliced to spliced mRNAs of each gene^18^. We applied the scVelo software, which uses heterogeneous splicing kinetics of different cell populations for the recovery of directed dynamic information^19^. The mRNA of all genes forms a multidimensional vector pointing in the direction of the overall increment of mRNA abundance of the cell. It thus predicts the future state of the cell transcriptome. In the gene space as provided by SOM, the mRNA velocity of a metagene is given by the multidimensional vector with the mRNA velocities of all single genes included in the metagene cluster^20^. It forecasts the expression change of the metagene. The cell or metagene-related vector fields were then transformed into trajectories of RNA velocity pointing in the direction of increasing expression. They consequently represent flow vectors of increasing mRNA abundance from a source toward a sink (or attractor) region of transcription.

## Results

### Treatment groups are divided into 7 types of cell activity

**Figure 2:**
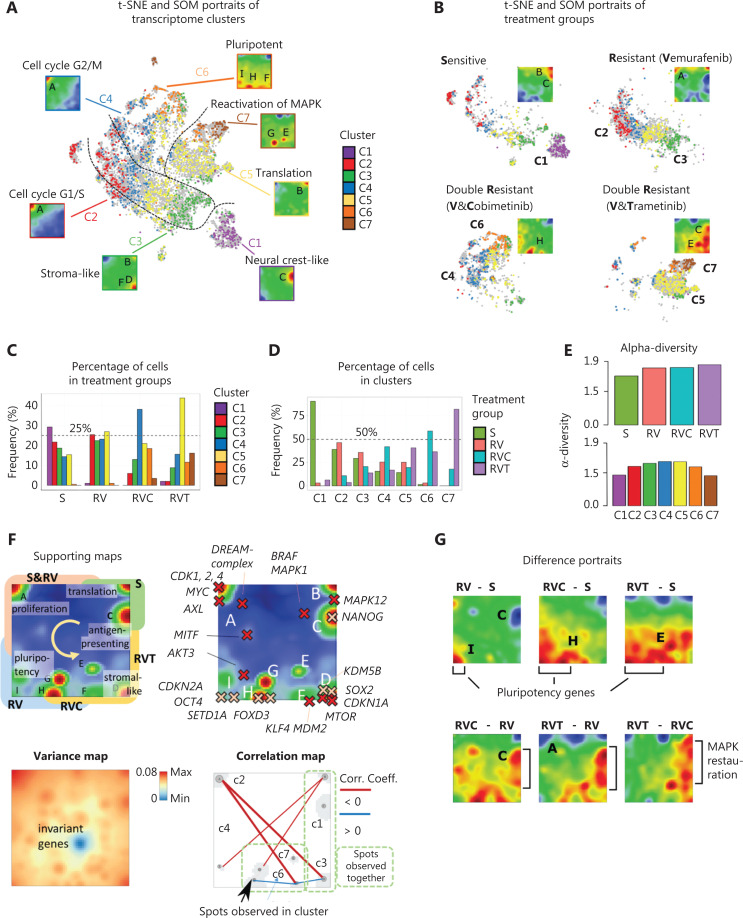
Single-cell RNA-seq of treatment sensitive (S), resistant (RV), and double-resistant (RVC and RVT) cell cultures. (A) t-Distributed stochastic neighbor embedding (t-SNE) overview of diversity of cell transcriptomes after clustering cells in 7 clusters, C1–C7. Mean expression portraits of each cluster reveal specific expression patterns as red-colored spot modules of upregulated genes indicated by capital letters A–J. The dashed curves in the t-SNE plot divide regions enriched in cells of the different treatment groups. (B) t-SNE and self-organizing map (SOM) portraits divided by treatment group. Specifically upregulated spots and populated clusters are indicated. The dashed curves separating the groups were taken from (A). (C) Distribution of cells in clusters among treatment groups. (D) Distribution of treatment groups among the clusters. (E) The alpha diversity of individual clusters and treatment groups was calculated in log scale as an entropy measure using the fractions of cells in the respective group^22^. (F) Supporting maps illustrate different properties of SOM space: The overexpression summary map provides an overview of the red spots observed in the individual group portraits (labels A–I) and their main function. Spot activation after treatment (S, RV, RVC, and RVT) follows the arrow pointing in a counterclockwise direction. Localizations of selected key genes are associated with the functional classification of the spots. The expression variance map indicates that the highest variability is observed along the edges of the map while invariant genes accumulate within the blue spot in the center. The correlation map shows strong anti-correlation of spot expression along the upper-left to down-right diagonal (between spots A and D). (G) Difference SOM portraits with respect to the untreated S-state indicate areas of differentially upregulated and downregulated genes in red and blue, respectively.

We applied scRNA-seq to measure the transcriptomes of a total of 26,979 single cells obtained from the A375 melanoma cell line grown under 4 different conditions: untreated/sensitive (S), treatment-resistant to BRAF inhibitor vemurafenib alone (RV) or treatment-resistant to a combination of vemurafenib with the MEK1/2 inhibitors cobimetinib (RVC) or trametinib (RVT) (**Figure 1**). Diversity analysis revealed 7 cell clusters of different transcriptomic activity (C1–C7), where each cluster is represented by a specific area on a t-SNE plot (**Figure 2A and Table 1**). Each cluster represents a transcriptional state of the cells characterized by its average SOM portrait of gene expression showing specific red areas (overexpression spots) of high transcriptional activity and blue areas (underexpression spots) of low transcriptional activity (**Figure 2A**).

**Table 1:** Functional characteristics of cell types

Cluster (cellular subtype)	Brief characteristics	Upregulated spots	High population in	Activated gene signatures^a^	Activated genes^b^
C1	Neural crest-like	C	S	Antigen-presenting cells, transitory neural-crest signature	*MAJIN*, *NME9*, *CD74*, *ERBB3*, *RAMP1*, *HLA-DRA*, *SOX10*, *KRT23*
C2	High cycling G1S-arrest biased	A	RV	Proliferating cells, high-cell-cycle activity, *DREAM* targets	*UBE2S*, *KRT81*, *KRT17*, *HMGN2*, *ANKRD1*, *FOSL1*, *KRT18*, *RANBP1*, *STMN1*, *CENPU*, *CDK1*, *CDK4*, *MYC*
C3	Slow cycling, stromal-like	D (B)	RV > (RVT > RVC)	Hallmark p53 targets stroma-like, epithelial-to-mesenchymal transition, cell cycle inhibition by CDKN1A	*NEAT1*, *CDKN1A*, *KDM5B*, *IL1B*, *H1-2*, *GSN*, *FAM3C*, *MTOR*, *MDM2*
C4	High cycling, G2M-arrest biased	A	RVC (> RV, RVT, S)	Proliferating cells, cell cycle activity in G2/M arrest	*See C2*
C5	Translation	B	RVT (> RV, RVC, S)	Ribosome, translation, low proliferation, melanoma housekeeping genes	*S100A6*, *RPL7A*, *RPL31*, *RACK1*, *EEF1A1*
C6	Stressed, pluripotency-resembling	F, H, I	RVC (> RVT)	Unconventional PI3K/Akt activators, KLF4 activation	*UTS2*, *COX2*, NTS, *MMP1*, *EGR1*, *MALAT1*, *SUCNR1*, *PCSK6*, *NEAT1*, *OCT4*, *SETD1A*, *KLF4*
C7	MAPK reactivating	E, G	RVT	Alternative MAPK pathway, stress-induced signaling, cGMP activity to MAPK, invasiveness	*EPO*, *MARCKS*, *TSPAN8*, *GUCY1B1*, *PDE6B*, *DDX10*, *BRAF*, *WDR63*, *SPANXD*

^a^Gene sets were taken from an number of earlier reports^8,21,46–48^ as part of the gene sets implemented in the R package oposSOM used for analysis^14^.

^b^Genes were taken from spot lists (**Supplementary Table S1**).

Each treatment group consists of several transcriptional clusters (**Figure 2B**), reflecting the heterogeneity of melanoma cells under each treatment^21^ (**Figure 2C**). S cells mainly consist of clusters C1–C5. Single-agent treatment-resistant RV cells mainly consist of clusters C2–C5, while double-agent-resistant RVC cells show a clear preference for C4, with less cells in C3, C5, and C6. Double-agent-resistant RVT cells were enriched by C5, followed by C4, C7, and C6 (**Figure 2C**). C1 largely consisted of S cells, thus harboring a cell type virtually eradicated by treatment (**Figure 2D**). In contrast, C6 and C7 almost exclusively consisted of double resistant cells from RVC and RVT, respectively, which defines them as specific double-agent treatment survivors (**Figure 2D**). The remaining clusters, C2–C5, constitute transcriptional cell states existing under all treatment conditions, but with different frequencies. Alternative similarity analyses such as independent component analysis (ICA) (**Supplementary Figure S1A**) and similarity network presentation (**Supplementary Figure S1B**) supported these results.

The heterogeneity of the treatment groups was translated into alpha-diversity values, an entropy-based measure derived from ecology to estimate diversity of species among habitats (**Figure 2E**)^22^. The alpha-diversity values among the treatment groups increased from sensitive toward RVT double-agent-resistant cells, reflecting the fact that heterogeneity of cell states increases upon treatment. In line with **Figure 2D**, the alpha-diversity among clusters is lowest in C1 and C7, which contain mainly S and RVT cells, respectively (**Figure 2E**). The high diversity among treatment groups supports the developmental character of transcriptomic clusters. Taken together, treatment resistance is reflected by a re-distribution of existing, and appearance of new gene expression clusters.

### Treatment affects gene patterns in a serial order

The red spots in the gene expression portraits (SOM) in **Figure 2A and 2B** consist of patterns of co-regulated genes overexpressed in the respective cell cluster or treatment groups, respectively. Overall, we identified 8 such spots labeled A–I (**Figure 2F and Supplementary Table S1**). Locations of selected key genes with functions in cell cycle activation, MAPK pathway, and pluripotency are indicated on the map (**Figure 2F**). Interestingly, the development of treatment resistance from sensitive (S), *via* single-agent-resistant (RV) toward double-agent-resistant (RVC/RVT) cells is reflected by an activation of spots from the top-right *via* top left toward the bottom right corner of the SOM, in counterclockwise direction, around an area of invariant genes. This leads to anti-correlated expression especially between genes in spot A and D (**Figure 2F, right**). Difference portraits of SOMs reveal that treatment activates new cell states with genes located along the left and lower edge of the SOM, while double-agent treatment resistance (versus single-agent treatment resistance) activates genes along the lower and right edges, reflecting partly reactivation of initial states of untreated cells in C1 (**Figure 2A and 2G**).

### Functional context of gene modules and cell types

**Figure 3:**
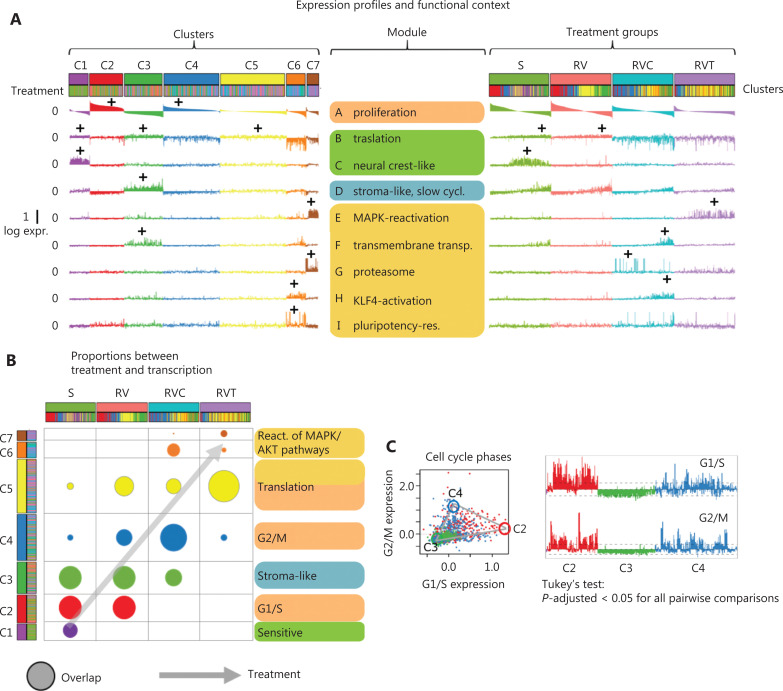

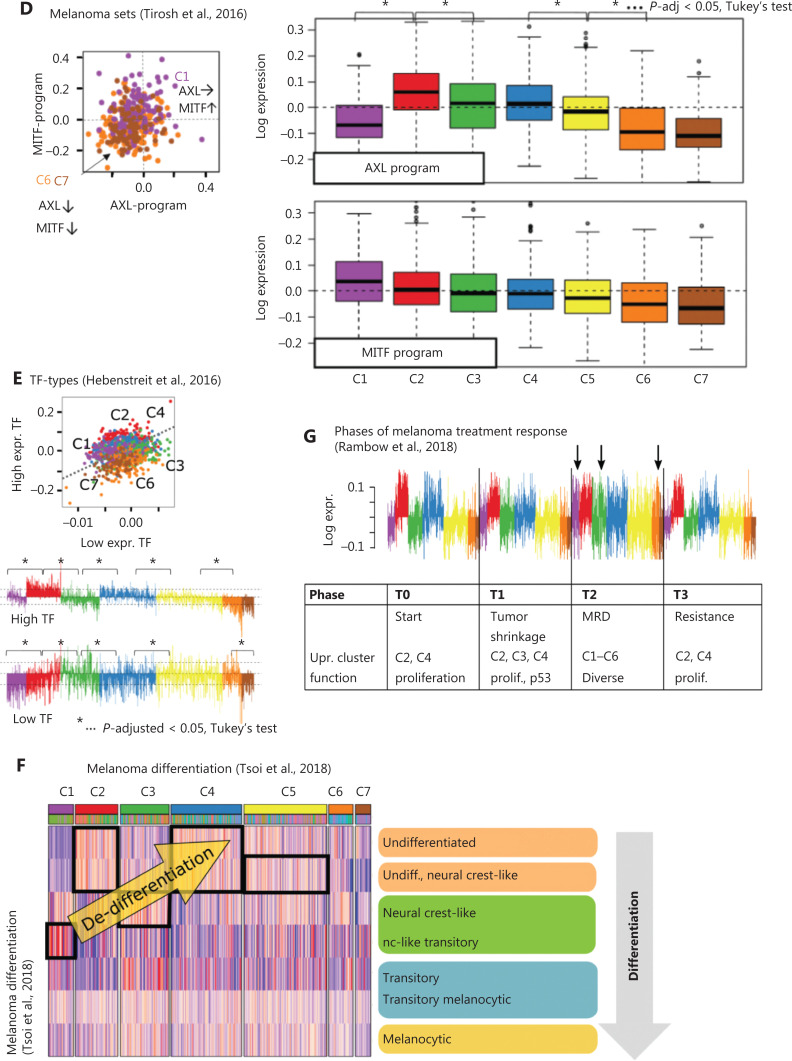
Expression profiles and functional context. (A) Profiles of self-organizing map spots A–I show mean log expression of genes included in the spots in all cells along the clusters (left) and treatment groups (right). Cells are sorted with decreasing expression of spot A (proliferative activity) in each cluster. (B) Mutual enrichment of cells in the cell type clusters and treatment groups. The arrow indicates the major trend observed upon treatment. (C)–(E) Pairwise comparison of expression signatures. (C) Comparison of gene expression in G1/S and G2/M phases of the cell cycle indicates a shift of C2 cells toward high expression of G1/S gene and of C4 cells toward high expression of G2/M genes, while C3 shows low expression of cell cycling genes. Red, blue, and green circles arranged along a triangle schematically illustrate the respective archetypal cell types. (D) Expression of AXL and MITF, respectively, gene expression programs in clusters C1–C7. (E) Expression of gene targets of low- and high-expression transcription factors (TFs) reveals expression bias toward high TF in C1, C2, and C4 and low TF in C3, C6, and C7^32^. (F) Heatmap of melanoma development gene signatures taken from Tsoi et al.^33^ mapped onto C1–C7 cluster signatures. Neural crest-like transitory signature genes are overexpressed in C1; genes characterizing undifferentiated melanoma cells are overexpressed in C2; and transitory (between neural crest and melanocytic) signature genes are overexpressed in C3. (G) Gene expression signatures taken from time courses after melanoma treatment in a mouse xenograft model activate cells of cluster C1, C3, and C6 at the minimum residual disease (MRD) state at T2 (arrows), which marks the transition between sensitivity and resistance for treatment^34^. Asterisks denote significant differences (*P* < 0.05, analysis of variance with Tukey’s post-hoc test).

Expression profiles of the genes from the different spots across all the cells are shown in **Figure 3A**, where cells were grouped by either clusters or treatment and ranked within each line with decreasing expression of spot A. The functional context of the spots was inferred from gene set analysis and/or location of selected key genes in or near the respective spots (**Supplementary Table S1**). The overlap plot of clusters and treatment groups provides an overview of the relationship between the 2 types of classifications (**Figure 3B**).

C1 is characterized by a specific upregulation of spot C, which is enriched with genes found in antigen presentation (MHC-II activation) and neural crest-like cells (**Table 1**). Key genes in the MAPK pathway, *BRAF*, *MAPK1*, and *MAPK12*, are located near spot C (**Figure 2F and Table 1**). After MAPK-inhibitor treatment, cells expressing spot (module) C mainly in C1 are lost (**Figure 3A**). Cells in C2 and C4 are marked by activation of spot (module)A, which includes genes involved in proliferation, mitosis, and stemness, all related to high proliferation, such as *MYC* and its targets and the cyclin-dependent kinases *CDK1* and *CDK4* (**Figure 3A and Table 1**). Comparing the activation of spot A among treatment groups reveals that RVT cells, and to a lesser extent RVC cells, are less proliferative than the other groups (**Figure 3A**). Interestingly, proliferating, partly de-differentiated S and RV cells are found in C2, while RVC cells are mainly found in C4 (**Figure 3B**). To differentiate better between C2 and C4, we examined the expression of genes involved in the G1/S and G2/M phase of the cell cycle, respectively, and found a shift from G1/S in C2 to G2/M in C4, while cells in C3 show low cycling activity (**Figure 3C**). Cells in C3 upregulated mainly spot (module) D, which enriches *p53* targets, signature genes of the epithelial–mesenchymal transition, stroma-associated genes, and *KDM5B*, an epigenetic marker of slow cycling in melanomas known to associate with drug resistance (**Figure 3A, Table 1 and Figure 2F**)^23,24^. *KDM5B* targets are known to drive a slow cycling state of melanoma after treatment-induced de-differentiation^25^. Furthermore, C3 and C5 cells show activation of genes encoding ribosomal proteins related to translation in spot B (**Table 1**).

All treatment groups have cells in C5, with the double-agent-resistant RVT cells showing the largest enrichment (**Figure 3B**). C6 clusters, and to a less extent C4 clusters, are specific for double-resistant RVC cells characterized by activation of spots (modules) F, H, and I. These spots contain genes related to pluripotency (*OCT4*, *SETD1A/KMT2F*, *KLF4*, or *FOXD3*) (**Figure 3A and 3B, Table 1**), which suggests a loss of differentiation of the respective cell subtype and acquisition of transcriptional plasticity. In melanoma, *KLF4* (spot H) (**Figure 2F**) plays a pro-tumorigenic and pro-proliferative role, inhibits apoptosis, and promotes metastasis in connection with ER-stress^26,27^. Other upregulated genes in C6 indicate activation of the PI3K/Akt pathway, inflammation, and metastasis (**Table 1**). Cluster C7 (mainly RVT cells) partly reactivates spot C (antigen presenting cells), but is mainly distinguished by spots E (*MAPK* reactivation) and G (proteasome) (**Figure 3B and Table 1**). Cells in C7 further express a variety of genes implicated in malignant processes, such as treatment resistance (*EPO*), proliferation *via* the JAK/Stat pathway, or invasion (e.g., *MARCKS*) (**Table 1**). Taken together, the functional patterns of cell clusters and SOM spots suggest that treatment resistance activates cell proliferation and de-differentiation, epithelial-mesenchymal transition, pluripotency, and MAPK reactivation.

### Pathway activation

Gene patterns of clusters C1–C7 were mapped onto 4 canonical signaling pathways (MAPK, JAK-STAT, p53, PIK3-AKT; **Supplementary Figures S2–S5**) using the signal flow (PSF) approach for estimating gene activity in the pathways^28^. This analysis showed that activation (in C2 and C4) and deactivation (in C3) of cell cycle genes appeared to be governed by downregulated or upregulated *CDKN1A* (cyclin-dependent kinase inhibitor), and the p53 antagonist *MDM2*, all involved in melanoma cell cycle de-regulation (**Supplementary Figures S2, S4, and S5**)^2^. A similar antagonism between *CDKN1A* and cyclin-dependent kinases in melanoma cell lines has been reported previously^29^. Moreover, reactivation of *MAPK*, *p53* and PI3K/Akt pathways, e.g., *via* activated protein synthesis was observed in C7 and, to a lesser degree, in C6 as a result of double-agent treatment (**Supplementary Figures S2, S4, and S5**). Thus, changes in gene expression under treatment appear to involve various intracellular signaling pathways.

### Epigenetics and comparison with previously described signatures of treatment resistance

Next, we assessed how our cell subtyping is related to previously published melanoma gene signatures. As shown in **Figure 3D**, the A375 cell line showed an upregulated AXL program in C2–C5, according to signatures taken from an earlier melanoma single-cell report and returned to baseline in C6 and C7^8^. The MITF program decreased during treatment resistance (**Figure 3D**)^8^. To estimate the possible effect of epigenetic factors in treatment resistance, we used expression data of chromatin states taken from genomic data of melanocytes (**Supplementary Figure S6D**)^30,31^. The proliferative clusters C2 and C4 showed enhanced expression of genes with active promoters and actively transcribed genes (TssA, Tx), indicating that proliferating A375 cells use canonical transcriptional programs of melanocytes, while genes with repressed and poised promoters (TssP, ReprPC) remain at low expression levels (**Figure 3E and Supplementary Figure S6D**). The latter states are, however, activated in double-resistant cells (RVT and RVC) suggesting their epigenetic de-repression after epigenetic remodeling as a possible mechanism promoting treatment resistance (**Figure 3E**). In support of this, we found a separation of clusters into 2 groups, C1, C2, C4 versus C3, C6, C7, respectively, regarding expression of high- and low-transcription factor (TF) characteristics (**Figure 3E**)^32^. The high-TF genes are associated with regulation of proliferation under TF-control, whereas low-TF genes refer to partly repressed genes under epigenetic control. Heatmaps of gene expression profiles of clusters C1–C7 and SOM spots are shown in **Supplementary Figure S6**.

Tsoi et al.^33^ reported a consecutive de-differentiation of melanoma cells upon resistance formation, which follows a trajectory from melanocytic *via* neural crest-like toward undifferentiated cells. We found a similar trend in RV, RVC, and RVT, namely that neural crest-like-resembling C1 cells virtually disappeared while the relative amount of cell types C2–C5, expressing different characteristics of undifferentiated cells, increased (**Figure 3F**). The fact that Tsoi et al. described a significantly longer time line (up to 90 days) of treatment, until development of resistance, might be the reason that we only observed a subset of de-differentiation states^33^. Furthermore, the A375 melanoma cells used in the present study originate from a long-term melanoma cell culture and will therefore have already undergone a degree of de-differentiation. This may explain the lack of expression of signatures of differentiated melanocytic cells in our data.

Signatures of de-differentiation were also found in single-cell analyses of a melanoma xenograft mouse model of treatment resistance to the BRAF inhibitor dabrafenib^34^. Regressing tumors in this model went into a state of minimum residual disease (MRD) indicating early treatment resistance (**Figure 3G and Supplementary Figure S6**). In our data, the MRD signature was characterized by activated C3 (stromal-like, slow cycling), C1 (neural crest-like), and C6 (pluripotency-resembling) cell types, supporting the role of de-differentiated cells in C3 and C6 for development of treatment resistance. Taken together, a number of known melanoma-related signatures were found to be activated in a cell subtype-specific manner in the present study during treatment resistance. We found different levels of support for our data by directly mapping expression signatures found in cell lines, animal models, and clinical data onto our data which confirmed their impact in a wider context beyond the particular cell line studied here.

### Pseudotime analysis reveals different treatment-dependent trajectories

**Figure 4:**
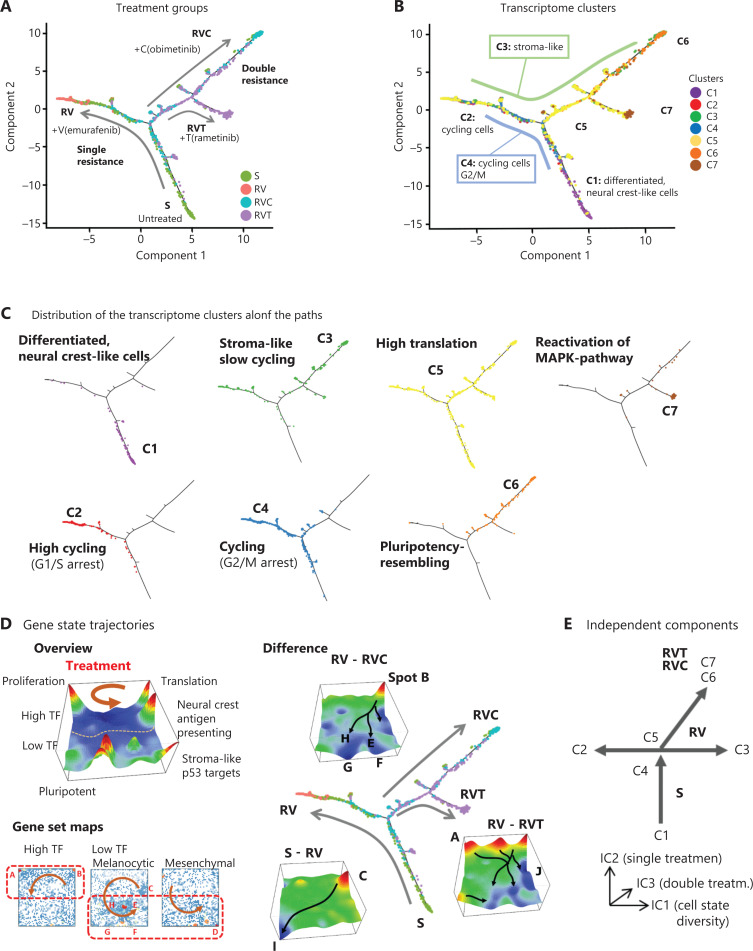
Tree analysis in cell and gene space. (A) Tree analysis using the pseudotime analysis program *monocle* reveals 3 treatment-dependent paths of either single-drug (RV) or double-drug resistance (RVC and RVT). (B, C) Coloring of cells according to transcriptome clusters C1–C7. (D) Sequential activation of spots in counterclockwise direction, where the upper/lower part of the self-organizing map (SOM) accumulate high-TF/low-TF genes (left). Areas of low-TF accumulation overlap with melanocytic and mesenchymal-related gene sets taken from a report of Wouters et al.^35^. Difference SOM portraits (right) between different treatment groups reveal differentially upregulated and downregulated gene spots (red and blue color, respectively). Note that blue activation patterns after double-drug treatment are similar for RVC and RVT. (E) Schematic representation of paths of resistance analyzed by independent component analysis.

Next, single-cell transcriptomes were analyzed by multibranched similarity trees using the *monocle* software to identify possible trajectories of treatment resistance^16^. The resulting pseudotime trees showed 3 branches, where the major backbone consisted of the transformation between S and RV cell states (**Figure 4A**). The side branches accumulated double-agent treatment-resistant cells with a split into 2 final states of RVC and RVT cells, respectively. Coloring of cells according to their cluster memberships provided further details about the developing cell states along the branches (**Figure 4B**). The starting point is represented by neural crest-like S cells (C1), and the 3 endpoints are represented by cycling RV (C2) and de-differentiated double-drug resistant RVC (C6) and RVT (C7) cells, the latter both showing low expression of cycling and translation-related genes (**Figure 4C**). G2M-arrested cells (C4) link C1 with C2 along the S-to-RV branch, suggesting that G2M-biased activation precedes G1S-biased activation in acquiring resistance. The area covered by C4 includes the split between single- and double-drug resistance branches where stromal-like cells (C3) are present along both sides, thus suggesting their involvement in the development of single- and double-drug resistance (**Figure 4B**). C5 cells are related to ribosomal gene expression and are distributed along all branches that supports enrichment of melanoma housekeeping genes, as taken from Tirosh et al. in this cluster (**Table 1**)^8^. Together, well-separated developmental trajectories are demonstrated after single- and double-agent treatment resistance. We also applied *URD*, an alternative, diffusion-based algorithm for reconstructing multibranched developmental trajectories, which yielded similar results (**Supplementary Figure S7**)^17^.

The cell subtypes are distributed widely along the different paths, which may reflect a cell state continuum. The description of changing cell states upon development is directly linked to changing gene states in transcriptomic landscapes^20^. Treatment resistance was associated with sequential activation of SOM spot expression in the transcriptional landscape, supported also by difference SOMs (**Figure 4D**). For example, SOMs show a shift from high- to low-expression transcription factor (TF) programs (**Figure 4D, lower**)^35^. ICA revealed that the observed cell subtypes are distributed along 3 independent components of variation (**Figure 4E and Supplementary Figure S1A**). Overall, pseudotime analysis in cell and gene expression space consistently defined clusters C1, C2, C6, and C7 as markers of cell subtypes dominating in the 4 treatment groups S, RV, RVC, and RVT, respectively. They are connected with each other *via* transitory-state cell types characterized by low (C3, C5) or disturbed (C4) proliferation.

### RNA velocity analysis of transcriptional plasticity upon treatment

**Figure 5:**
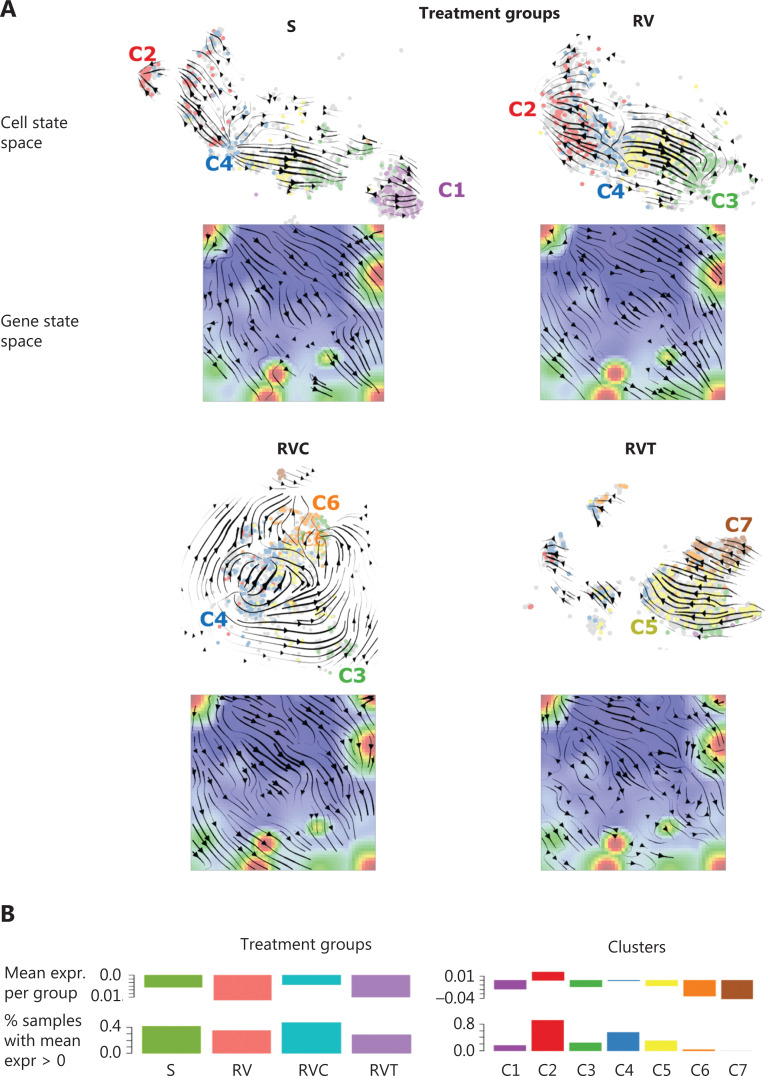
RNA velocity dynamics in cell and gene state space. (A) RNA velocity vector fields in t-Distributed stochastic neighbor embedding (t-SNE; cell space) and self-organizing map (SOM; gene space) plots for the different treatment groups. A part of the groups (C1, C2, C3) are sinks of vectors (arrow heads pointing toward these cell clusters), whereas other groups (C4, C5) are sources (arrow heads pointing away from these clusters). In the gene space, the vector field points from the left upper corner (high proliferation) toward the right lower part (low proliferation) part of the SOMs. (B) Total expression values in treatment groups (left) and transcriptome clusters (right) either per cell (ranked with increasing expression), per group, or as number of cells exceeding a threshold.

RNA velocity analysis offers an independent approach for studying developmental dynamics^18^. It calculates the change of mRNA abundance in every single cell and uses this to predict its future transcriptional state^18,19^. In t-SNE cell state space, this analysis provides vectors for all cells that are summarized into a trajectory field pointing in the direction of the development of the cells under the different treatment conditions (**Figure 5A**). Cluster C4 (G2M cycling cells) forms a sort of source for trajectories pointing in the direction of C2 (G1S cycling cells) and C3 (stroma-like, slow cycling cells) under untreated conditions (S) or single-agent treatment resistance (RV). Cluster C4 points toward C6 or C7 of double-agent treatment resistance (RVC and RVT). This result is in agreement with the pseudotime trees, where C4 cells are accumulated near the branching points, leading to the different cell subtypes at their ends, namely C2, C6, and C7 cells (**Figure 4**). RNA velocity dynamics further supports the view that these cell clusters represent stable attractor states under the different treatments. Stable attractors under untreated conditions are C1 (neural crest) and C2 (proliferation) cells. In contrast, C4-type cells appear to form a transient state, which tends to further develop into the other types. C3 cells represent attractors under single-agent treatment (RV), but a transient state under the other conditions.

The RNA velocity concept was also applied to gene state space (SOMs), where RNA velocity indicates to the rate change of mRNA expression in each metagene-pixel of the SOM (**Figure 5A**)^20^. The velocity vectors in untreated (S) and single-agent-resistant (RV) cells point away from spot A (cell cycle) in the left upper part of the map toward spots C (neural crest) and D (stroma) in the right part of the SOM. This is in agreement with the source characteristics of spot A expressing C4 cells as well as the attractor characteristics of C1 and C3 cells in S and RV. Trajectories of double-agent treatment resistance point toward the regions related to pluripotency and epigenetic reprogramming (in RVC) as well as MAPK pathway reactivation (RVT), in the lower part of the SOMs. Especially in the latter case, trajectories were disturbed by local attractor regions, which possibly reflects a more plastic transcriptional landscape.

RNA velocity estimates directional alterations of mRNA abundance, which are expected to be related to total expression levels (**Figure 5B**). High overall expression was observed in highly proliferating (C2 and C4) and translating (C5) cells referring to the high-expression transcription factor (TF)-regulated cells, while C3 (stroma-like) and C6/C7 cell types were on relatively low expression levels (**Figure 5B**).

Taken together, RNA velocity analysis provided support for developmental paths induced by single- and double-agent treatment resistance. Interestingly, the G2M cycling cells in C4 form a bifurcation-like transcriptional state at the crossroad toward treatment resistance acquired in C3, C6, and C7.

## Discussion

**Figure 6:**
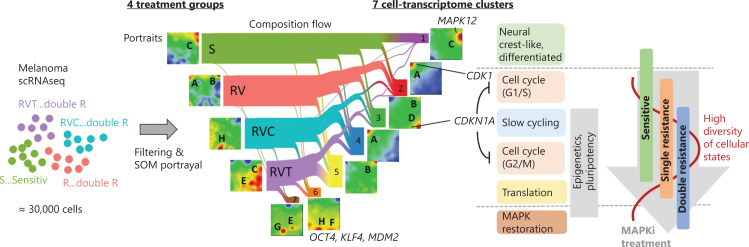
Single-cell trajectories of melanoma resistance to mitogen-activated protein kinase inhibitor treatment. Single-cell RNA-seq data were generated from 4 different groups of A375 cells (S, RV, RVC, AND RVT). Their self-organizing map portraits show specific spot patterns, which were grouped into 7 cell clusters, C1–C7. The composition-flow presentation shows how cells in the different treatment groups are composed of the cell-type clusters. C1 and C6/C7 are specific for sensitive (S) and double-agent-resistant (RVC and RVT) cells, respectively, while C2–C5 correspond to cell subtypes present under all conditions but at varying levels of enrichment. Maximum diversity of cellular states is observed after single treatment (RV).

The present single-cell transcriptomic study describes the diversity of cellular states and trajectories of phenotypic changes of A375 melanoma cells during the development of treatment resistance to targeted therapy. Overall, 7 cellular states described as clusters C1–C7 were discernable under the 3 conditions of treatment resistance (RV, RVC, RVT) and the untreated reference state (S). The gene expression landscape of each state is characterized by its specific SOM portrait (**Figure 6**). About 30% of MAPK inhibitor-sensitive cells belong to cluster C1, consisting of antigen-presenting cells with neural crest-like gene expression patterns. The remaining 70% of cells were equally distributed among 4 different clusters, C2–C5, which consist of highly cycling (C2 and C4) cells and slowly cycling stroma-like (C3) and translationally active (C5) cells. Under single-agent (vemurafenib) resistance, C1 cells almost completely disappeared. In contrast, the other cell states, C2–C5, were enriched and formed a reservoir of heterogeneous, treatment-resistant cellular states. Thus, a loss of differentiated neural crest-like cells and enrichment of highly proliferative, stroma-like and undifferentiated phenotypes appears to be an initial feature of single-agent treatment-resistant melanoma cells. Tumor plasticity per se, i.e., the ability of cancer cells to dynamically switch between different cell differentiation states, represents an important oncologic process that can be shaped by drugs. Sensitivity includes total eradication of cell states such as C1, but also changes in the relative amounts of C2–C7 due to the heterogeneous effects of treatment on the fitness of these cell states. C6 and C7 clusters presumably arise because of specific survival advantages in double-agent-resistant RVC and RVT cells and are related to MAPK reactivation and pluripotency.

Support for a de-differentiated phenotype under BRAF-inhibitor treatment resistance came from an earlier single-cell study^36^. Melanoma cells under BRAF inhibitor treatment developed signatures of neural crest stem cells and epithelial-to-mesenchymal transition, expressing genes associated with elevated invasiveness and migration^36^. In a subsequent study, 2 different trajectories of BRAF inhibitor resistance were characterized by either a proliferative cell state and NGFR/AXL expression or MITF/MART expression^37^. These findings are in line with our observations showing that highly proliferative clones are major components of single-drug (RV) and double-drug (RVC) resistant clusters. De-differentiation of melanoma cells has also been described in another report, based on signatures derived from melanocyte differentiation programs^38^.

In a recent study using single-cell technology, the authors showed melanoma cell lines and patient specimens to be composed of a series of transcriptionally distinct states, consistent with the results reported here^39^. Importantly, the cell state composition was dynamically regulated in response to BRAF inhibitor therapy, and the transcriptional state composition predicted therapy response. The differences in fitness between the transcriptional states were relevant and informative for the optimization of therapy schedules to retain the drug-sensitive population. These findings might argue for the use of an intermittent treatment modality in which drug withdrawal permits the re-emergence of sensitive cells, possibly rendering the tumor treatment-sensitive again. However, clinical investigations supporting these findings are missing.

Tsoi et al.^33^ defined different developmental states in melanoma cell lines based on bulk transcriptome sequencing. Resistance trajectories after BRAF inhibition started either as pigmentation or neural-crest like cell types and ended up as de-differentiated cell types and partly overlapped with proliferation patterns. In line with this, the A375 vemurafenib-sensitive (RV) cells in our experiments started as neural-crest-like transitory cells on their trajectory and passed *via* a stromal and a highly proliferative, de-differentiated cell type under single-drug treatment to treatment resistance.

It has been shown that slow-cycling cells expressing the marker gene *KDM5B* (a histone H3K4 demethylase, also termed *JARID1B*) were involved in long-term melanoma growth^24^. *KDM5B* expression was also observed in resistant clones in a study from Shaffer et al.^40^. Recent research revealed that expression of *KDM5B* follows a highly dynamic equilibrium across melanoma cells^24^. When challenged with drugs, the intrinsically slow-cycling *KDM5B* high expression cell state became initially enriched, whereas under persistent drug-exposure melanomas decrease *KDM5B* expression again to re-enter cell proliferation for long-term tumor repopulation. Accordingly, *KDM5B* appears to represents a checkpoint for coordinating differentiation of melanoma cells *via* transcriptional reprograming, and cell cycle delay. In line with this, we found that the *KDM5B*-high cell state C3 (spot D) was located at the crossroads between sensitive/single-agent treatment resistant to double-agent treatment-resistant groups. Hence, it is located at a pivotal position along the developmental trajectories leading to treatment resistance, which is in line with the above-mentioned findings. Interestingly, the cyclin-dependent kinase inhibitor *CDKN1A* was co-expressed with *KDM5B*, which suggests cooperation between modes of transcriptional regulation, governed by transcription factors and epigenetic mechanisms upon developing treatment resistance. Such cooperation is a hallmark of stem cells and argues for a stem-like patterns, in combination with pluripotency, leading to treatment resistance in melanoma cells^41^. Pluripotency is a characteristic of stem cell-like populations, and pluripotency transcriptomic patterns are found to make up a significant proportion of double-agent treatment resistant cells in the present study (C6). Moreover, in the present study, the increased diversity of cellular states after treatment was associated with activation of pluripotency markers such as *OCT4* and *KLF4* (C6 cells), which suggests loss of differentiation and development of cellular plasticity. This trend is further supported by the enrichment of targets of so-called low expression transcription factors, reflecting epigenetic regulation, e.g., *via* changing the histone code^32^.

Tumor resistance was found to be associated with extracellular matrix deposition in an earlier single-cell study of a murine model of BRAF-mutant melanoma^42^. In the present study, stroma-like cell phenotypes were observed especially in C3 cells, which were distributed over all treatment groups. Thus, extracellular matrix re-organization appears to be a mechanism of treatment resistance to targeted treatment. In line with this, fibroblast signatures have been shown to impact on immune cell infiltration *via* expression of different complement factors as shown in the above mentioned single-cell melanoma study^8^. Single-cell trajectories as derived from pseudotime analyses of a xenograft mouse model for melanoma treatment with the BRAF inhibitor dabrafenib showed that an early proliferative state developed *via* 2 different developmental trajectories into 2 types of highly proliferative stem cells, namely neural crest-like and starved-like melanoma cells, which are enriched in a minimal residual disease (MRD) state^34^. These signatures showed analogies with C1-, C3-, and C6-type cells in our study.

MAPK reactivation appears to be a common feature of melanoma treatment resistance to double-agent treatment as shown in earlier DNA or RNA sequencing studies, in which mutational mechanisms such as amplifications of mutant *BRAF*, mutations in *NRAS*, and mutations in phosphoinositide kinase (PI3K)/Akt pathway were common drivers of pathway reactivation^43–46^. In the present study, MAPK pathway reactivation was observed in C7 (RVT) cells at late stages of pseudotime trajectories on a transcriptional level. These findings underline the impact of treatment resistance driven by transcriptional reprogramming. In one of these studies, transcriptomic patterns in single- and double-drug-treated disease progressors, compared with patient-matched baseline melanoma tissues, indicated upregulated gene expression of tumor and stromal genes^45^. Among the top downregulated genes were genes involved in antigen presentation (B2M, HLA-A, HLA-B, and TAP1). This finding is in line with our observation of a loss of the C1 cluster (antigen-presentation) in treatment resistance.

## Conclusions

The cellular plasticity of cancer cells and phenotypic heterogeneity remain a major challenge for the treatment of melanoma and other types of cancer^47,48^. In the present report, it is shown that melanoma cells have the capacity to switch their phenotype under treatment from a differentiated phenotype to a more de-differentiated phenotype. Single-cell transcriptomes of melanoma cells revealed insights into responses to targeted treatment on a cellular level. SOM portraits provided the underlying transcriptomes with single-cell resolution. Trajectories of treatment resistance were characterized by transcriptional patterns of cellular proliferation, de-differentiation, and slow-cycling stromal states, finally leading to patterns of pluripotency and MAPK reactivation, partly driven by epigenetic mechanisms. This heterogeneity of cellular states appears to be crucial for developing treatment resistance. These findings argue for a continuous development of treatment resistance in a dynamic equilibrium of cellular states, with possible consequences for future treatment options targeting early markers of the de-differentiation process. These new options may include substances directed against AXL kinase, mitochondrial inhibitors targeting slow-cycling cells, or glutathione peroxidase 4 (GPX4) inhibitors targeting de-differentiated cells states, as previously described^48^. Together, targeting cellular plasticity represents a promising option for future treatment approaches.

## Data Availability

Data are available in the GEO database (GSE164897).
