# Supplementary material for: Single-cell trajectories of melanoma cell resistance to targeted treatment
Source: Cancer Biol Med. 2021 Oct 1;19(1):56–73. doi: 10.20892/j.issn.2095-3941.2021.0267 (PMC8763000; doi:10.20892/j.issn.2095-3941.2021.0267)
Supplement: Supplementary file 1 [file cbm-19-056-s001.pdf]

Supplementary materials

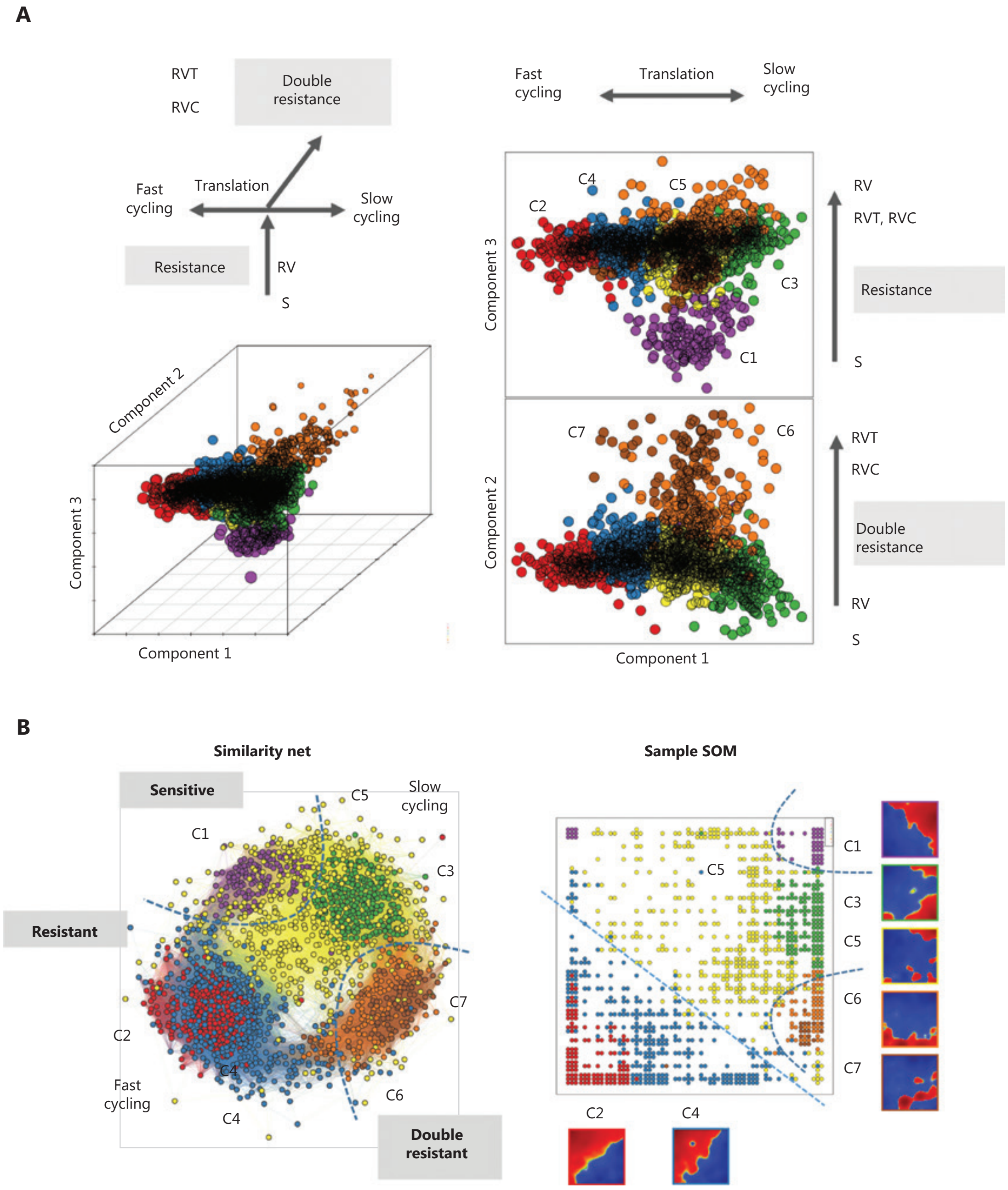

Figure S1 Sample similarity analysis. (A) Independent component analysis (ICA) of the single-cell transcriptomes revealed that treatment shifts cells along component 3 (compared with untreated S-cells), whereas double-agent treatment shifts cells along component 2 (compared with single-agent treatment). Heterogeneity of cell states, especially in RV cells, distributes cells along component 1. ICA was performed as implemented in oposSOM after stratification of cells into clusters C1–C7[1]. (B) Similarity net and sample SOM provide 2 alternative options to visualize similarity relations between cells[2]. Despite different method-specific geometries, both methods show mutual correspondence of the similarity relations of cell clusters C1–C7. Largest variance is observed for C2–C5, mostly referring to single-agent-resistant cells (RV), while C1 (sensitive, S) and C6–C7 (double resistant RVC and RVT) cells occupy localized areas in the plots. Along the edges of the sample SOM, cluster-specific expression portraits are shown. They are colored in the so-called log-log scale, which shows overexpression in red and underexpression in blue; thus, the borderline between both regions was a sort of coastline. The methods are described in detail in oposSOM[1].

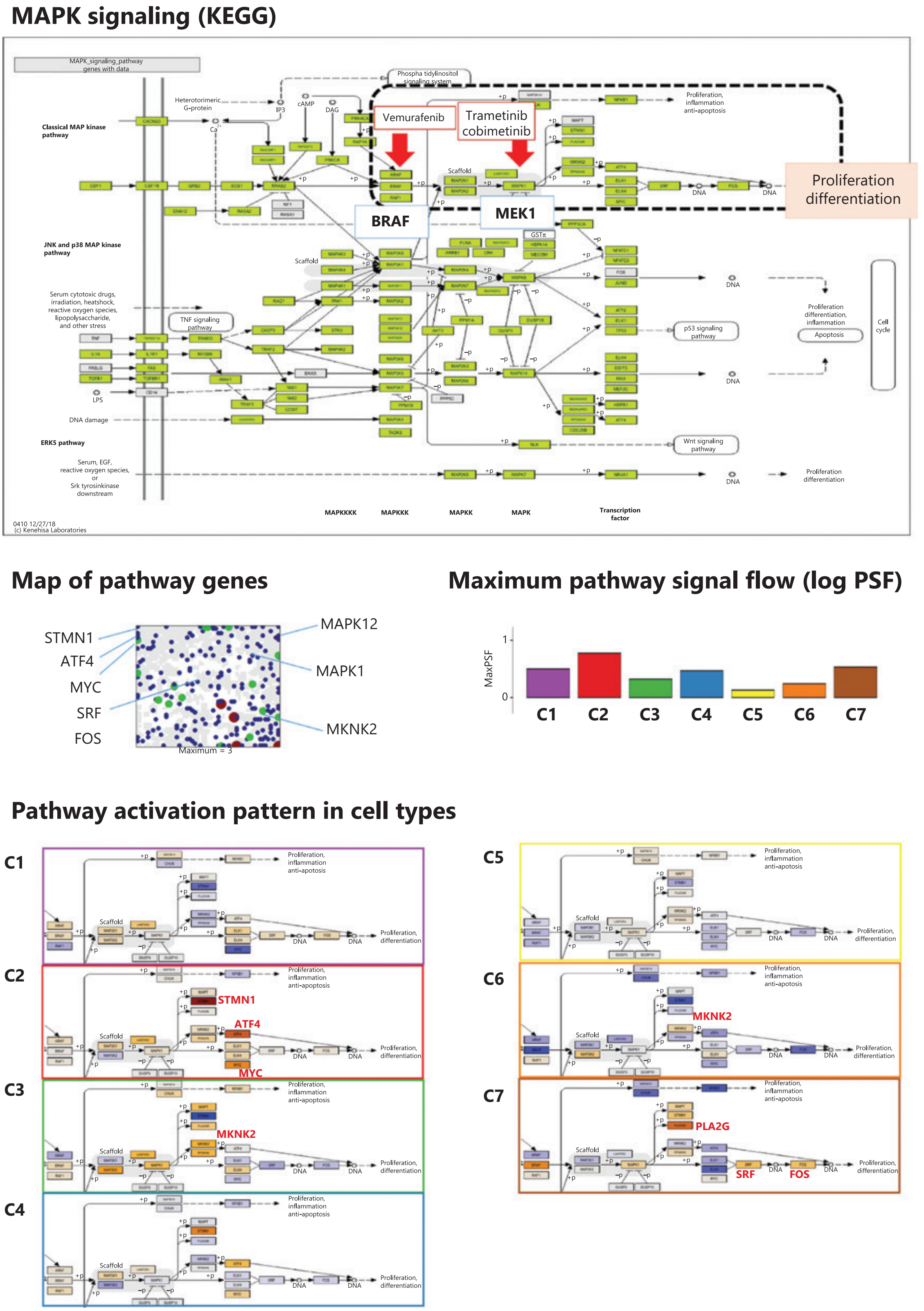

Figure S2 MAPK signaling pathway as provided by the Kyoto Encyclopedia of Genes and Genomes (KEGG) and implemented in oposSOM[1]. The red arrows in upper panel mark modes of action of the BRAF inhibitor vemurafenib and of MEK1 inhibitors cobimetinib and trametinib along the MAPK cascade. The MAPK pathway consists of nodes (mostly genes) and edges providing (activating or deactivating) interactions

in a left-to-right direction. The pathway signal flow (PSF) analysis (middle panel) of the MAPK signaling pathway estimates the activity of the genes based on their expression values[3]. The color of the nodes range from blue (low PSF activity) to brown (high activity) (lower panel). For example, activation of proliferation is indicated by STMN1 and ATF4 expression in C2 and, to a lesser degree, in C4. Representative pathway activities in the different cell types were estimated using the maximum PSF value in any node.

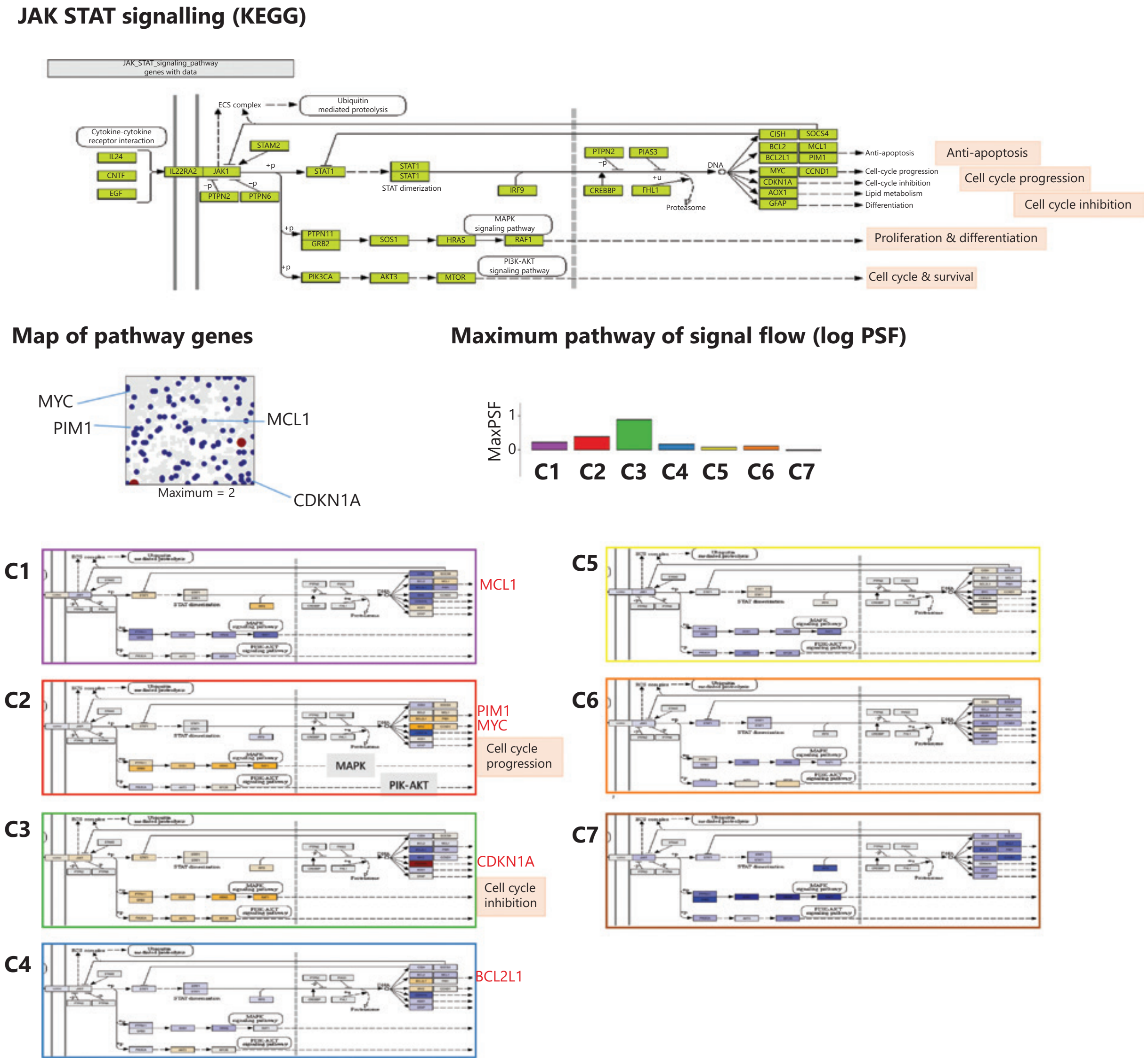

Figure S3 JAK STAT signaling pathway as provided by the Kyoto Encyclopedia of Genes and Genomes (KEGG) and implemented in oposSOM[1]. Analyses were performed as described in **Figure S1**. PSF-analysis of JAK-STAT signaling shows maximum activity in C3 cells that was associated with expression of the *CDKN1A* gene located in the right lower corner of the SOM (middle panel), upregulated in C3-type cells. In the other cell types, CDKN1A remains at a low expression level. In C2, and partly C4, MYC activation promotes cell cycle progression. JAK-Stat signaling appeared deactivated in double-agent-resistant C6 and C7 cells (lower right panels).

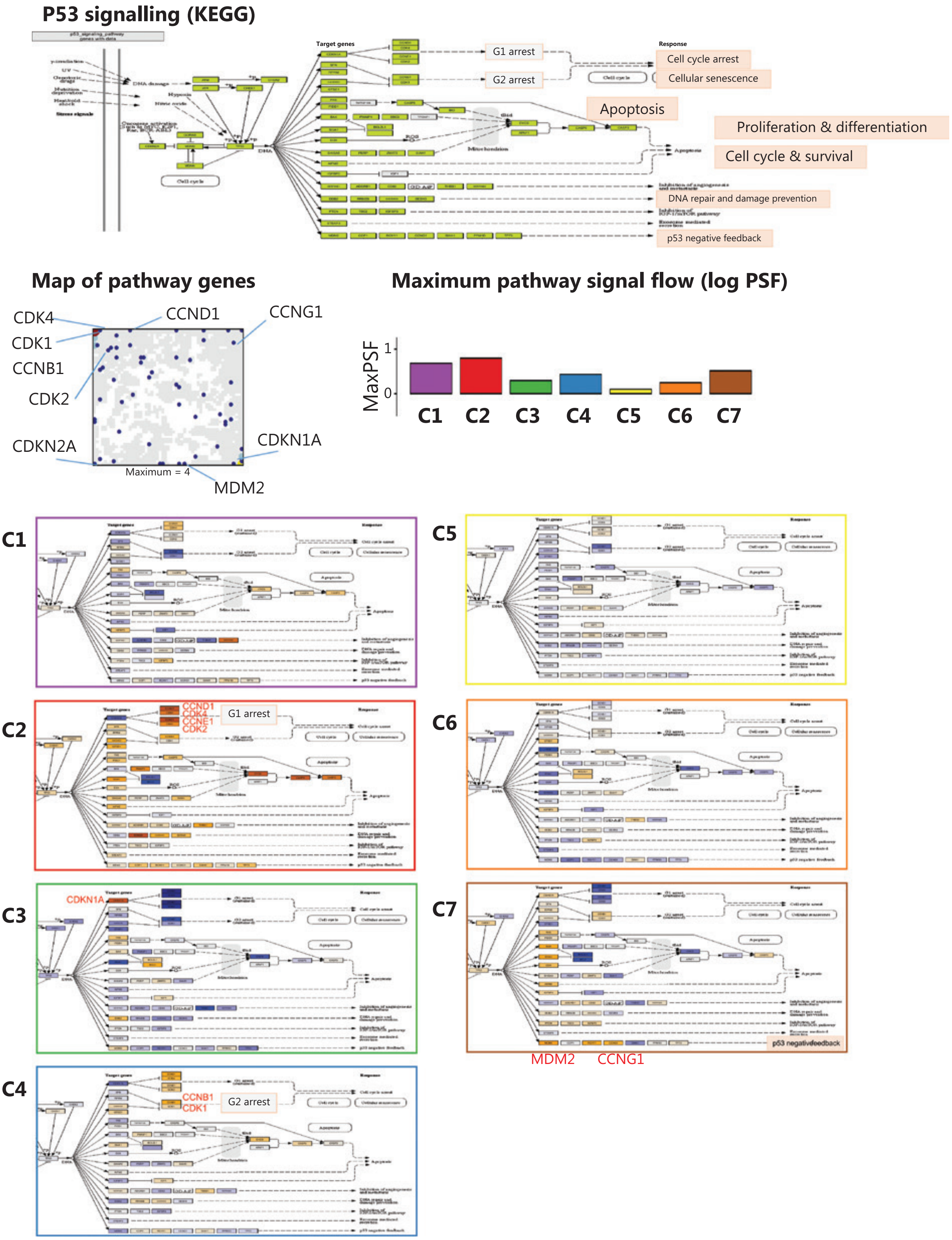

Figure S4 P53 signaling pathway as provided by the Kyoto Encyclopedia of Genes and Genomes (KEGG) and implemented in oposSOM[1]. Analyses were performed as described in **Figure S1**. PSF-analysis of p53 signaling shows activation of cell cycle arrest in C2 and C4. Cell cycle arrest is biased toward the G1S phase of the cell cycle in C2 and toward the G2M phase of the cell cycle in C4. In C3, the p53 pathway is inactivated, and cell cycle arrest is mediated *via* cell cycle inhibitor CDKN1A. The p53 pathway is reactivated in C7.

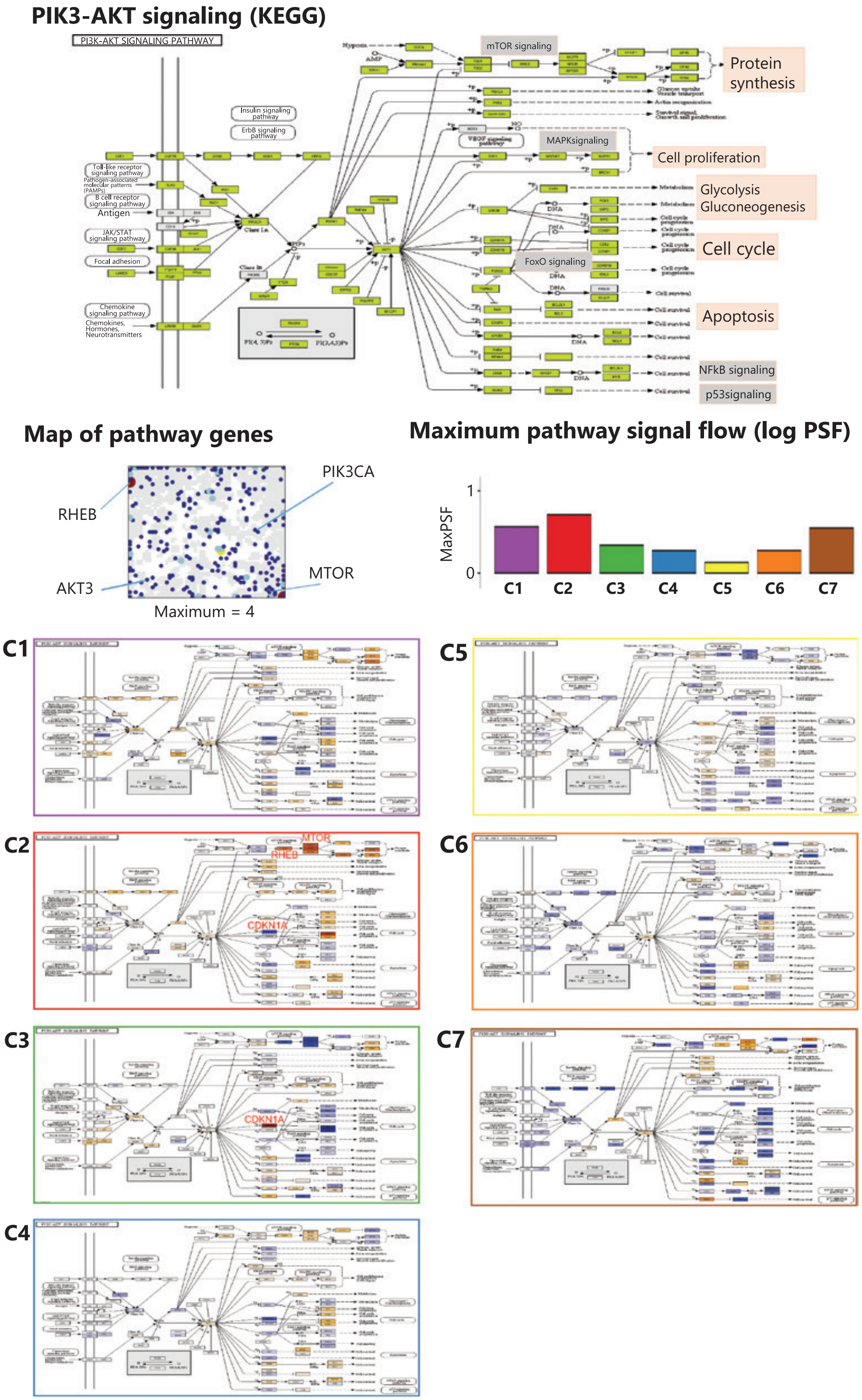

Figure S5 PIK3/AKT signaling pathway as provided by the Kyoto Encyclopedia of Genes and Genomes (KEGG) and implemented in oposSOM[1]. Analyses were performed as described in **Figure S1**. PSF-analysis of PIK3/AKT signaling indicates activation in C1, C2, and C7 with activation of the MTOR complex.

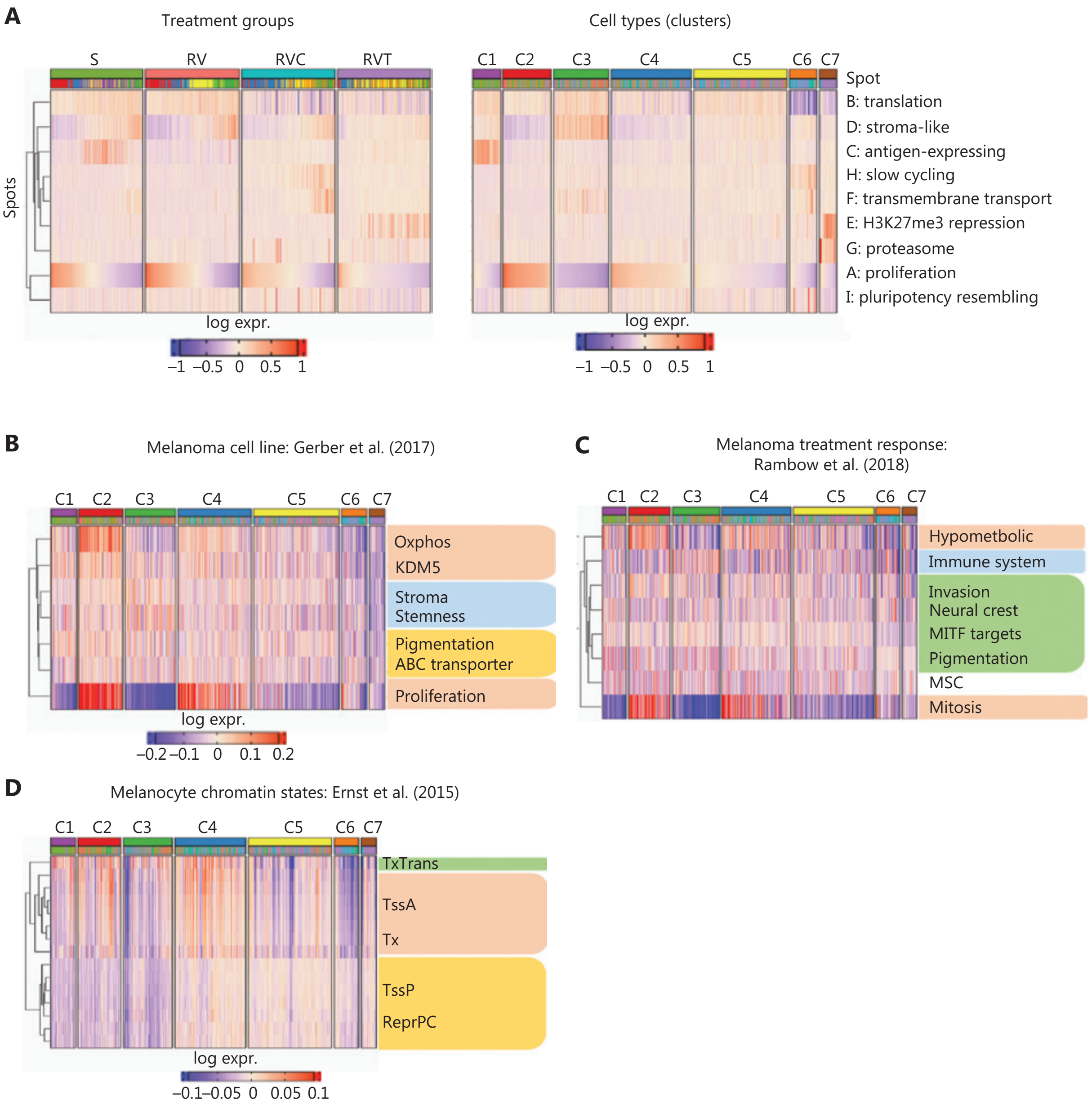

Figure S6 Heatmaps of gene expression profiles. (A) Heatmaps of expression profiles of SOM spots A–I in the treatment groups (left) and cell clusters (right). Cells are sorted with decreasing expression of spot A (proliferative activity). (B) Single-cell melanoma signatures from patient-derived cell lines indicate proliferative activity in C2 and C4, oxidative phosphorylation (oxphos) in C2, and stemness/stromal-like characteristics in C3[4]. (C) Signatures of melanoma treatment response taken from a single-cell melanoma study of Rambow et al.[5]. (D) Gene expression in A375 cells of genes stratified according to different chromatin states described by the Roadmap Epigenomics Consortium and by Ernst et al.[6,7]. Actively transcribed (TxTrans, Tx) genes in melanocytes and genes with active promoters (TssA) are highly expressed in C2, C4, and, partly, C1, suggesting that these genes retain their chromatin state compared with melanocytes. In contrast, expression of these genes decreased in C6, and partly in C3. Expression of genes from poised (TssP) and repressed (ReprPC) promoters are expressed especially in C6, suggesting remodeling of chromatin.

Supplementary Results

Multibranched developmental trajectories reveal a bottleneck between sensitive and resistant cell states (Figure S7)

For independent tree analysis in pseudotime scale (0 < PT < 1), we applied *URD*, a diffusion-based algorithm for reconstructing multibranched developmental trajectories[8]. URD distributes the cells along 3 branches after treatment (b1–b3) enriched with RV (branch 1, b1), RVC (b2), and RVT (b3) cells, respectively (**Supplementary Figure S7, lower**), which supports the results obtained using *monocle* presented in the main text. The mean SOM portraits along the 3 branches indeed resemble that of the different treatment groups (**Figure 2B, Supplementary Figure S3**). Accordingly, b1 represents RV and C2 (high proliferation) cells, especially at pseudotime values greater than 0.3, while b2 and b3 represent RVC and RVT (C6 and C7), respectively. At earlier pseudotime (PT < 0.3), the composition of cells is more diverse and includes C5 (high translation) and C3 (stroma-like) (both in b2 and b3) and C4 (G2M cycling cells, in b1). Interestingly, alpha-diversity runs through a minimum at PT ~ 0.3, reflecting the transition from sensitive to resistant cell types *via* a bottleneck of low cell diversity. As pre-bottleneck transitory states, we identified C4 (G2M-arrested cycling cells), C3 (stroma-like), and also C5 (activated translation/housekeeping genes), whereas post-bottleneck resistant states are C2 (G1S-arrested cycling cells in b1 treated with RV), C6 (pluripotent RVC), and C7 (MAPK-reactivated RVT).

In summary, the development proceeds from differentiated cells *via* transitory states (translation, stroma/slow cycling, G2M-arrested) toward final G1S-arrested (RV) to single- and double-agent-resistant cells (RVC, RVT).

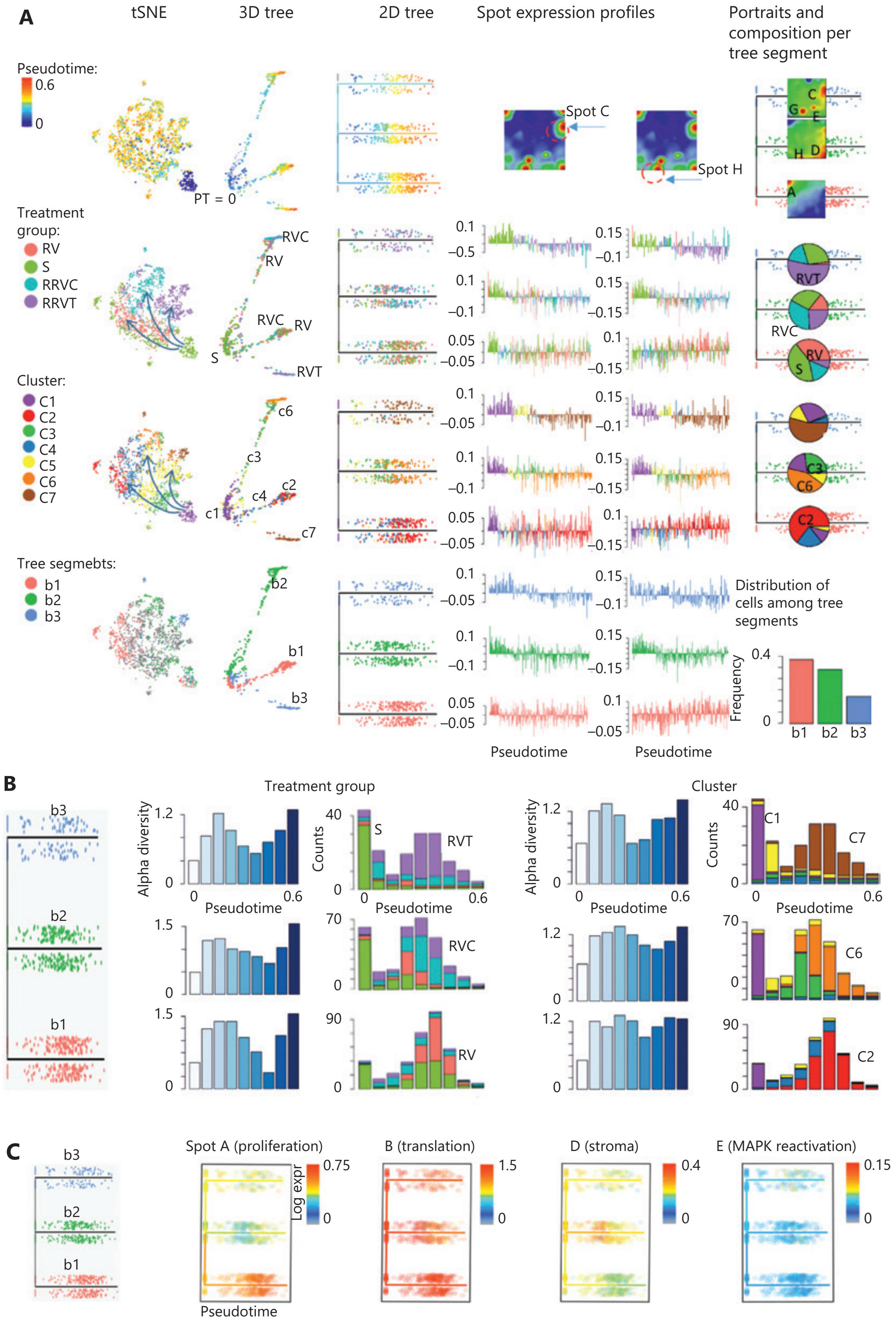

Figure S7 Pseudotime analysis of developmental paths of treatment resistance using *URD*[8]. (A) t-SNE, 3D (3 dimensional) and 2D *URD* trees provide different views on cell-similarity space where cells were colored according to pseudotime (0 < PT < 1), treatment, transcriptome cluster membership and tree branches (b1, b2, and b3) (rows A–D, respectively). Selected spot profiles show their expression in cells as a function of PT. Branches were characterized by the mean SOM expression portraits, composition of cell strata (percentages of cells in treatment groups

and clusters, see pie diagrams) and number of cells included (bar plot below). (B) Alpha diversity runs through a minimum, indicating a diversity bottleneck between early and later phases of developing treatment resistance (bar plot at the right). Plots of composition as a function of PT show that treatment-resistant cell patterns arise at later PT > 0.3. (C) Trees colored according to expression of spots A, B, D, and E.

Table S1 SOM spot characteristics

SOM spot	Brief characteristics	Up in cell type (s)	Up in	Gene sets and *P* value of enrichment[a]	Activated genes[b]
A	Proliferation	C2, C4	S, RV	FISCHER_DREAM_TARGETS 1e-99 Pujana_CHEK2_network 2e-99 WONG_EMBRYONIC_STEM_CELL_CORE 1e-76 HALLMARK_E2F_TARGETS 1e-67 HALLMARK_MYC_TARGETS_V1 1e-59 BenPorath_Proliferation 1e-52 Tirosh_Core_cycling_genes_in_melanoma 2e-70	635 genes *CCNA2*, *MYC*, *CENPA*, *ESPL1*, *ARHGEF28*, *HMMR*, *ADRB2*, *NMU*, *NGFR*, *STC2*, *CDK1*, *CDK4*
B	Translation melanoma housekeeping	C1, C3, C5	S, RV	KEGG_RIBOSOME 1e-99 BP Viral_transcription 1 e-99 MF translation 4e-89 Tirosh_housekeeping_genes 1e-51	295 genes *RPL32*, *RPL7A*, *ULBP2*, *CMTR1*, *FARP2*, *SMAD4*, *FAS*, *EIF3F*, *MORC3*, *FAP*
C	Neural-crest-like; antigen presenting	C1	S	CC clahrin_coated_endocytic_membrane 1e-08 BP MHC class II protein complex 1e-07 BP antigen processing and presentation 2e-06 CC endocytic vesicle membrane 1e-04	653 genes *SLX1B*, *MAJIN*, *NPFFR2*, *HLA-DRB5*, *HLA-DRB1*, *HLA-DQA1*, *S100B*, *ZBTB8B*, *SOD3*, *MADCAM1*
D	p53 targets, stroma-like slow-cycling	C3	S, RV	FISCHER_DIRECT_P53_TARGETS_META_ANALYSIS 5e-16 Lee_BMP2-targets_UP 1e-16 HALLMARK_P53_PATHWAY 1e-12 CC collagen-containing extracellular matrix 1e-09 CC extracellular exosome 1e-12	568 genes *CDKN1A*, *KDM5B*, *JAK3*, *WNT2B*, *SERPINF2*, *SOX6*, *SOX2*, *HRAS*, *JUND*, *IGFBP3*, *NFKB1*
E	MAPK reactivation	C7	RVT	*MAPK* genes: *MAPK-9*, *MAPK-11* Calcium-related: Ca-channel (*CACN* genes), calmodulin Protein phosphatases (*PPP3* genes)	162 genes *CCL27*, *GPC2*, *SLC23A3*, *HVCN1*, *ANXA8*, *PDGFRL*, *FGG*, *SIRT4*, *ZNRF3*, *TNXB*
F	Transmembrane transport; Slow cycling; c3 and RVC specific	C3, C6	RVC, S	WIRTH Mucosa 5e-05 BP transmembrane transport 1e-03	273 genes *RASL11B*, *VEGFD*, *CARD9*, *PINCR*, *BCL11B*, *GNG13*, *ITGAX*, *SVEP1*, *CES4A*, *SMARCD3*
G	Proteasome	C7	RVT	Reactome_Ubiquitination_proteasome 1e-05 KEGG ubiquitin_mediated_proteolysis 1e-04 Regulation of mitotic cell cycle 1e-04	271 genes *GSTM2*, *IL12RB1*, *RAB33B*, *TRIM22*, *RPH3AL*, *TAGLN*, *ABLIM3*, *SPANXD*, *IL12A*, *ITGB3*
H	KLF4 activated	C6	RVC	Wierenga_STAT5A_targets 1e-05 Reactome CTLA4_inhibition 1e-04 BP keratin filament 1e-03	427 genes *UTS2*, *CPT1B*, *DLX3*, *SUCNR1*, *TFCP2L1*, *FOXD3*, *PPM1L*, *SERPINA3*, *KRT6A*, *KRT13*, *KLF4*
I	Pluripotency-resembling	C6, C5, C4, C3	RVC, RV	BP Negative_regulation_of_immune_response 1e-09 Benporath ES-with_H3K27me3 1e-03 Tirosh_AXL−signature 1e-03	627 genes *OCT4*, *HES4*, *DVL1*, *MMP23B*, *EGLN3*, *GNAZ*, *HRH4*, *PIWIL2*, *PIWI1L*, *RHOV*

[a]Gene sets were taken from a number of earlier studies as part of the gene sets implemented in the R-package oposSOM[1,3,6,8-10]. Enrichment *P* values refer to Fisher's exact test.
[b]Activated genes were selected as examples from the spot modules. Full lists are provided as **Supplementary Table S2**.

References

1. Löffler-Wirth H, Kalcher M, Binder H. oposSOM: R-package for high-dimensional portraying of genome-wide expression landscapes on bioconductor. Bioinformatics. 2015; 31: 3225-7.
2. Hopp L, Wirth H, Fasold M, Binder H. Portraying the expression landscapes of cancer subtypes. Systems Biomed. 2013; 1: 99-121.
3. Nersisyan L, Löffler-Wirth H, Arakelyan A, Binder H. Gene set- and pathway-centered knowledge discovery assigns transcriptional activation patterns in brain, blood, and colon cancer. Int J Knowledge Discov Bioinformatics. 2014; 4: 46-69.
4. Gerber T, Willscher E, Loeffler-Wirth H, Hopp L, Schadendorf D, Schartl M, et al. Mapping heterogeneity in patient-derived melanoma cultures by single-cell RNA-seq. Oncotarget. 2017; 8: 846-62.
5. Rambow F, Rogiers A, Marin-Bejar O, Aibar S, Femel J, Dewaele M, et al. Toward minimal residual disease-directed therapy in melanoma. Cell. 2018; 174: 843-855.e19.
6. Kundaje A, Meuleman W, Ernst J, Bilenky M, Yen A, Heravi-Moussavi A, et al. Integrative analysis of 111 reference human epigenomes. Nature. 2015; 518: 317-30.
7. Ernst J, Kheradpour P, Mikkelsen TS, Shoresh N, Ward LD, Epstein CB, et al. Mapping and analysis of chromatin state dynamics in nine human cell types. Nature. 2011; 473: 43-9.
8. Farrell JA, Wang Y, Riesenfeld SJ, Shekhar K, Regev A, Schier AF. Single-cell reconstruction of developmental trajectories during zebrafish embryogenesis. Science. 2018; 360: eaar3131.
9. Liberzon A, Birger C, Thorvaldsdóttir H, Ghandi M, Mesirov JP, Tamayo P. The Molecular Signatures Database (MSigDB) hallmark gene set collection. Cell Syst. 2015; 1: 417-25.
10. Subramanian A, Tamayo P, Mootha VK, Mukherjee S, Ebert BL, Gillette MA, et al. Gene set enrichment analysis: a knowledge-based approach for interpreting genome-wide expression profiles. Proc Natl Acad Sci USA. 2005; 102: 15545-50.
